# Ionizing Radiation-Induced Oxidative Stress in Computed Tomography—Effect of Vitamin C on Prevention of DNA Damage: PREVIR-C Randomized Controlled Trial Study Protocol

**DOI:** 10.3390/jcm13133866

**Published:** 2024-06-30

**Authors:** Camilo G. Sotomayor, Camila González, Miki Soto, Nicolás Moreno-Bertero, Claudina Opazo, Baltasar Ramos, Gonzalo Espinoza, Álvaro Sanhueza, Gonzalo Cárdenas, Sebastián Yévenes, Jorge Díaz-Jara, José de Grazia, Marcia Manterola, Daniel Castro, Abraham A. I. J. Gajardo, Ramón Rodrigo

**Affiliations:** 1 Radiology Department, University of Chile Clinical Hospital, University of Chile, Santiago 8380420, Chile; 2 Anatomy and Developmental Biology Program, Institute of Biomedical Sciences, Faculty of Medicine, University of Chile, Santiago 8380453, Chile; 3 Faculty of Medicine, University of Santiago Chile, Santiago 9170022, Chile; 4 School of Medicine, Faculty of Medicine, University of Chile, Santiago 8380453, Chile; 5 Human Genetics Program, Institute of Biomedical Sciences, Faculty of Medicine, University of Chile, Santiago 8380453, Chile; 6 Intensive Care Unit, Medicine Department, University of Chile Clinical Hospital, University of Chile, Santiago 8380420, Chile; 7 Program of Pathophysiology, Institute of Biomedical Sciences, Faculty of Medicine, University of Chile, 8380453 Santiago, Chile; 8 Molecular and Clinical Pharmacology Program, Institute of Biomedical Sciences, Faculty of Medicine, University of Chile, Santiago 8380000, Chile

**Keywords:** computed tomography, ionizing radiation, oxidative stress, DNA damage, antioxidants, vitamin C

## Abstract

Exposure to ionizing radiation (IR) is inevitable in various X-ray imaging examinations, with computed tomography (CT) being a major contributor to increased human radiation exposure. Ionizing radiation may cause structural damage to macromolecules, particularly DNA, mostly through an indirect pathway in diagnostic imaging. The indirect pathway primarily involves the generation of reactive oxygen species (ROS) due to water radiolysis induced by IR, leading to DNA damage, including double-strand breaks (DSB), which are highly cytotoxic. Antioxidants, substances that prevent oxidative damage, are proposed as potential radioprotective agents. This *Study Protocol* article presents the rationale for selecting vitamin C as a preventive measure against CT-associated IR-induced DNA damage, to be investigated in a randomized placebo-controlled trial, with a full in vivo design, using an oral easy-to-use schedule administration in the outpatient setting, for the single CT examination with the highest total global IR dose burden (contrast-enhanced abdomen and pelvis CT). The study also aims to explore the mediating role of oxidative stress, and it has been written in adherence to the Standard Protocol Items recommendations.

## 1. Introduction

### 1.1. Ionizing Radiation in Medical Imaging: Computed Tomography and Patient Dose

**Figure 1:**
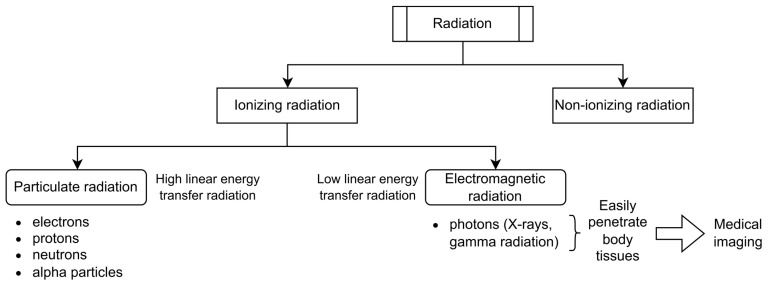
Classification of radiation, ionizing radiation, and examples including X-rays with its use in medical imaging.

Radiation encompasses any moving form of energy. It is categorized into ionizing and non-ionizing forms. Ionizing radiation (IR) is further classified into high and low linear energy transfer radiations. The latter, electromagnetic radiations (X-rays and γ radiation), travel as waves that easily penetrate body tissues [1,2]. In particular, X-rays have become increasingly used to reveal anatomy and internal organs for medical purposes (Figure 1).

The average background radiation remains steady at 3 millisieverts (mSv) per year and has shown minimal change over the past three to four decades [3]. However, total global per capita radiation exposure doubled until 2010, largely attributed to the increasing radiation from medical sources [3,4]. The escalating doses associated with computed tomography (CT) scans have been identified as the primary driver [3]. In the United States in 2016, 230 CT scans per 1000 people were conducted, with abdomen and pelvis scans comprising the largest category (~20 million) [5,6]. Yet, a recent global assessment (2009–2018) indicated a greater CT increase in other countries [5]. While annual background radiation is ~3 mSv, a whole-body CT scan exposes individuals to approximately 10 mSv [1,7]. 

For CT, radiation exposure is quantified with CT dose index-volume (CTDIvol) and dose-length product (DLP) [3]. However, they do not provide an accurate patient dose estimate [8,9] because they do not consider how much absorbed radiation dose biologically affects tissues [1]. Patient dose estimates need to consider standard-sized patient region-specific “k factors” [8,9,10,11,12], which can then be adjusted for specific patient size and shape [9,13,14,15]. The patient dose estimate termed effective dose (ED) is endorsed by the International Code of Radiology Practice for dosimetry in diagnostic radiology [16], as it was long developed by the International Commission on Radiological Protection [17].

Effective dose (ED) = DLP × *k* factor (corrected for specific-patient size)

DLP, scan region, and patient size enable the estimation of effective dose (ED), which is the preferred radiation quantification parameter for assessing the individual patient effects of CT-related IR exposure. For abdomen and pelvis CT, additional adjustments considering abdominal fat may enhance the precision of ED estimation [18].

### 1.2. Ionizing Radiation and DNA Damage: Indirect Mechanism through Oxidative Stress

**Figure 2:**
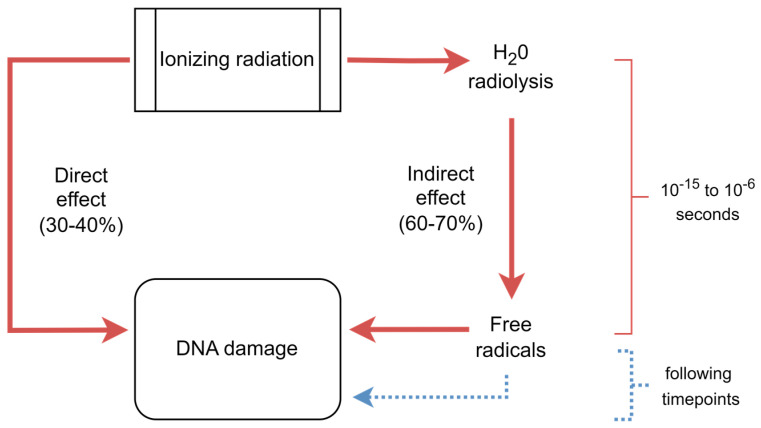
Direct (30–40%) and indirect effects (60–70%) of exposure to ionizing radiation lead to DNA damage.

The notion of genetic material impairment resulting from ionizing radiation (IR), such as chromosome breakage, precedes our comprehension of DNA’s structure [19]. Initially, a direct pathway implicating collisions between high-energy particles or photons and DNA strands was proposed. However, an oxidative stress (OS)-mediated indirect mechanism of IR-induced DNA damage was later described, accounting for the majority (60 to 70%) of the total DNA damage induced by IR [20,21,22,23,24]. OS occurs due to an imbalance between the production of oxidant species and the activity of the antioxidant system, which favors the first. This OS-mediated indirect mechanism was described as generating reactive oxygen species (ROS) via IR-induced water radiolysis (Figure 2).

Within a fraction of a second (10^−15^ to 10^−6^ s), IR promotes the release of electrons, triggering homolytic cleavage of covalent bonds, which generates free radicals and ROS. While the direct pathway leads to immediate DNA damage, and the indirect pathway leads to DNA damage both immediately and over varied timeframes, DNA damage occurring after the first second of post-exposure contributes to total DNA damage in a significantly minor proportion, with previous studies showing that DNA damage increases within the first 5 min post-exposure and thereafter it decreases [25]. This evidence is in line with the notion that experiments assessing DNA damage should take care to avoid repair of strand breaks [26], providing the rationale to propose that DNA damage induced by IR may be assessed with 5 min interval post CT. Some ROS are consumed in neutralizing reactions, but those present in the cellular nucleus often engage in propagation reactions, potentially leading to DNA damage, including double-strand breaks (DSBs) [20,21,22,23,24,27,28,29,30], which are considered the most relevant and cytotoxic lesions. If not repaired or repaired incorrectly, they can cause structural chromosomal abnormalities, possibly initiating mutations and thereby contributing to carcinogenesis [31,32,33]. Phosphorylation of the histone variant H2AX is one of the initial steps in the cellular response to DSBs. Hence, the immunofluorescence analysis of this phosphorylated histone, i.e., γ-H2AX, is widely used to monitor IR-induced DNA damage [34]. 

### 1.3. Antioxidant Defense System: Exogenous Antioxidants against IR-Induced Oxidative Stress

Antioxidants are substances that delay, prevent, or eliminate oxidative damage to target molecules [35,36,37]. Endogenous antioxidants serve as the primary defense against OS by effectively neutralizing ROS [38,39]. Under conditions of heightened ROS levels, however, endogenous defenses may be overwhelmed, resulting in OS [38,40]. In such cases, exogenous antioxidants can bolster overall antioxidant defense [38,41], being proposed as radioprotective agents through various mechanisms [28]. The mechanisms involved in IR-induced DNA damaging and repairing by different radioprotectants have been previously revised and detailed, with existing research suggesting that free radical scavenging and induction of enzymatic endogenous antioxidants may play a significant role against most of the IR-induced indirect DNA damage [37] (for a comprehensive overview on proposed effects and mechanisms of different radioprotective agents, we underscore the in-depth literature review article by Smith et al. [28]). Thus, biomarkers of the antioxidant capacity (e.g., ferric reducing ability of plasma; FRAP assay [42]) previous to exposure may be associated with IR-induced DNA damage. 

Vitamin C has been described as one of the most potent antioxidants and can prevent IR-induced DNA damage through direct and indirect antioxidant mechanisms [37,43,44]. Directly, it eliminates ROS due to its electron-donating capacity, which allows it to oxidize to dehydroascorbate [37]. At certain plasma concentrations, it also alters the reaction between the superoxide anion and nitric acid [45,46]. Indirectly, vitamin C inhibits ROS-producing enzymes and inflammatory responses via NF-κB pathway inhibition [38,47]. It prevents the OS-enhancing effect of endothelial nitric oxide synthase by stabilizing tetrahydrobiopterin [38,46,48]. It also enhances the effect of other antioxidants, such as recycling alpha-tocopherol [37,49]. A recent and growing body of evidence from experimental studies with different designs (i.e., in vitro, in vivo, or mixed in vitro/in vivo) supports vitamin C as a novel antioxidant intervention to reduce IR-induced DNA damage in patients undergoing CT imaging (Supplementary Materials) [50,51,52,53,54,55]. 

Beyond vitamin C, previous in vitro studies [52,55] have also compared different antioxidants, showing that vitamin C and NAC had the highest significant reduction of the excess DNA damage induced by IR. In the clinical setting [51], the effect of vitamin C was two-fold higher than that of NAC, even administered minutes prior to IR exposure, which is an important consideration under the light of time-setting data showing that the effect of NAC increases until its peak at minute ~30, with a steeper curve than vitamin C, which reaches its peak at minute ~60 (which provides a rationale to propose that supplementation strategies may maximize its impact at around 60 min post-intervention). Yet, aiming for wide clinical use and a high impact on total global IR dose burden, intravenous administration strategies may limit its clinical application. Moreover, it should also be noted that high-dose antioxidant strategies may lead to counterproductive effects. Whereas DNA protective effects may be observed at vitamin C plasma levels ~50 μmol/L [56], breakage-type chromosomal aberrations may be induced at ~110 μmol/L, of notice, even in non-irradiated cells [57]. Although low oral doses of vitamin C (~600 mg) have shown significant results in a mixed in vitro/in vivo study [53], consistent reduction of excess DNA damage induced by IR is found with intermediate oral doses of ~1 g. It should be underscored that no previous study has performed vitamin C measurements prior to (or after) the supplementation strategy, which is lacking to provide further understanding of how baseline vitamin C levels potentially influence result variability. Provided that low/high doses of vitamin C are avoided, further evaluation of hypothetical increased effects with the addition of other antioxidants could not be consistently provided with current evidence from clinical studies. Yet, a combination of antioxidants showed a 58% reduction of excess DNA damage [34], comparable to the 61% effect size found with intermediate oral doses of vitamin C [54], and in vitro studies have also failed to provide consistent evidence supporting additive effects [52].

Existing literature holds the plea for further investigation into the mediating role of OS previous to exposure in IR-induced DNA damage, particularly focusing on the outpatient setting, with the single CT examination with the highest total global IR dose burden (electromagnetic radiation, i.e., excluding particulate radiation), using the most accurate estimation methods for patient dose. The initial premise that OS plays a significant mediator role in CT-associated IR-induced DNA damage forms the basis for proposing the hypothesis that enhancing antioxidant capacity through an exogenous antioxidant intervention (1 g oral vitamin C, one hour prior to exposure) could offer new interventional opportunities.

## 2. Materials and Methods

### 2.1. Study Design

This Study Protocol adheres to the Standard Protocol Items (SPIRIT) guidelines [58]. It outlines a prospective interventional randomized placebo-controlled study conducted under blind conditions for both the assessors and the patients. The study will take place at the Radiology Department of the academic University of Chile Clinical Hospital and has received approval from the Medical Ethics Committee (AA 84/23 on 10 January 2024) and hospital Board (AA 02/24 on 24 January 2024). Certification from the Office for Clinical Research (AC 1399/24 on 25 January 2024) has been obtained. The Center Coordinators are designated with specific roles (C.G.S. is the Principal Investigator, and A.I.J.G. is the Internal Responsible Investigator), and a Steering Committee has been established comprising C.G.S., D.C., A.I.J.G., and R.R. Study protocol version identifier 1.0. Trial intended registry name: PREvention of Ionizing Radiation-DNA damage through oxidative stress in computed tomography with vitamin C (PREVIR-C)

### 2.2. Study Population and Recruitment

The study population comprises consecutive patients with health concerns related to the abdomen and pelvis organs and a clinical indication for contrast-enhanced (CE) abdomen and pelvis CT in the outpatient setting, with scheduled appointments at the Radiology Department of the University of Chile Clinical Hospital. Patients will be recruited via telephone call approximately 36–72 h before their appointment. Additionally, non-IR-exposed health-related personnel volunteers and their relatives without an indication of IR-associated examination will be recruited as a control group through a direct onsite invitation from the researchers.

**Figure 3:**
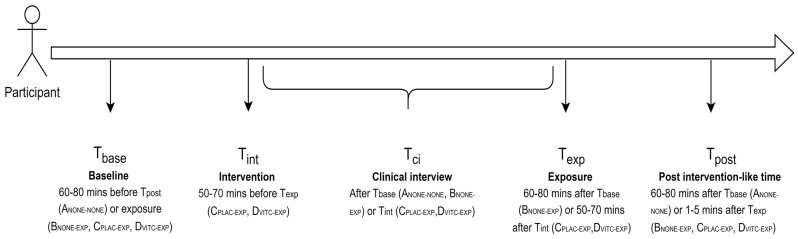
Study timeline. T_base_, baseline, informed consent signing and collection of blood sample #1, 60–80 min before collection of blood sample #2 (all groups). T_int_, intervention (groups C_PLAC-EXP_ and D_VITC-EXP_), 50–70 min before T_exp_. T_ci_, clinical interview, immediately after T_base_ (groups A_NONE-NONE_ and B_NONE-EXP_) or T_int_ (groups C_PLAC-EXP_ and D_VITC-EXP_). T_exp_, exposure, 60–80 min after Tbase (B_NONE-EXP_) or 50–70 min after T_base_ (group B_NONE-EXP_) or after T_int_ (groups C_PLAC-EXP_ and D_VITC-EXP_), with the collection of blood sample #2 immediately prior to CT for groups C_PLAC-EXP_ and D_VITC-EXP_. T_post_, post-intervention-like schedule, collection of blood sample #2 (groups A_NONE-NONE_ and B_NONE-EXP_) or #3 (groups C_PLAC-EXP_ and D_VITC-EXP_), 60–80 min after T_base_, ~1–5 min after T_exp_ for groups B_NONE-EXP_, C_PLAC-EXP_ and D_VITC-EXP_.

- Inclusion criteria: Male and female patients aged 18 to 75 capable of providing a signed informed consent.
- Exclusion criteria: Pregnant women, severe chronic kidney disease (glomerular filtration rate < 30 mL/min/1.73 m^2^), or contraindications for antioxidants supplementation or iodinated contrast examinations. Genetic syndromes, onco-hematologic diseases or history of peptic ulcers or urinary stones. Occupational exposure to IR; radiation therapy or chemotherapy in the last 6 months, or exposure to other IR-associated examinations in the previous 72 h or immediately after the abdomen and pelvis CT. Use CE CT premedication, antioxidant supplementation regularly or on the day of the CT exam, iron supplementation, or an iron-restricted diet.
- Sample size: proposed on the basis of other clinical studies to detect a significant reduction of increased DNA damage in the interventional group [50,51,52,53]. Assuming increases in γ-H2AX foci ranging 1–99% (~20% dispersion [59]), a sample size of *n* = 25 patients per group (assuming *n* = 3 missing values), there is an 80% power to detect any mean difference between the placebo and interventional groups > 16%.
- Study groups: Four groups, A_NONE-NONE_, B_NONE-EXP_, C_PLAC-EXP_ and D_VITC-EXP_, named according to corresponding intervention (none/placebo/vitamin C) and exposure (none/CE abdomen and pelvis CT), as detailed below. For reference on the study subjects’ protocol timeline, please also see Figure 3.
  - A_NONE-NONE_: Not recipients of any intervention (placebo or vitamin C) nor exposure (CE abdomen and pelvis CT). Blood sample #1 (T_base_) will be collected 60–80 min before blood sample #2 (T_post_), with a clinical interview in between (T_ci_).
  - B_NONE-EXP_: Not recipients of any intervention but exposed (T_exp_). Blood sample #2 (T_post_) will be collected 60–80 min after the blood sample #1, with a clinical interview (T_ci_) followed by the exposure (T_exp_) in between. Based on comparative data with A_NONE-NONE_ (i.e., interim estimates of the exposure effect), re-estimation of the starting sample size (*n* = 25) may be considered for these non-interventional groups [60]. With an estimation of the exposure effect, randomized placebo-controlled enrollment will start for the interventional (C_PLAC-EXP_/D_VITC-EXP_) groups.
  - C_PLAC-EXP_: Recipients of a placebo (T_int_) and exposed (T_exp_). Blood sample #2 will be collected immediately prior to CT. Blood sample #3 (T_post_) will be collected 60–80 min after blood sample #1, with the administration of the placebo (T_int_), clinical interview (T_ci_) and exposure (T_exp_) in between.
  - D_VITC-EXP_: Recipients of the oral vitamin C intervention (T_int_) and exposed (T_exp_). Blood sample #2 will be collected immediately prior to CT. Blood sample #3 (T_post_) will be collected 60–80 min after blood sample #1, with the administration of the vitamin intervention (T_int_), clinical interview (T_ci_) and exposure (T_exp_) in between.

### 2.3. Study Protocols

- Study subjects’ protocol timeline: according to the study points shown in Figure 3.
- Placebo/vitamin C intervention: Patients to be exposed (i.e., with an indication of CE abdomen and pelvis CT in the outpatient setting) to the interventional parallel groups **C_PLAC-EXP_** and **D_VITC-EXP_** will be randomly assigned to receive the one-time oral placebo or vitamin C intervention with a 1:1 ratio by means of computer-generated randomization (SPSS Inc., Chicago, IL, USA), 50–70 min before the CT exposure. The researcher will generate and assign treatment allocation without any interaction with study subjects (D.C.). Group **C_PLAC-EXP_** patients will be orally administered the placebo as effervescent tablets dissolved in 250 mL water. Group **D_VITC-EXP_** patients will be orally administered the vitamin C intervention (1 g) as effervescent tablets dissolved in 250 mL water. The placebo is prepared by the laboratory with the same presentation (package, color) and the same excipients of the active drug, organic acids (citric or tartaric) to simulate the acidity and an inert substance (starch or similar) to achieve the equivalent weight. The subjects and the researcher in charge of enrolment and placebo/vitamin C administration (C.G.S.) will be blind to treatment allocation. D.C. will reveal participants allocated to intervention only after the results of the primary and secondary outcomes have been.
- Two-phase CE abdomen and pelvis CT study protocol: All patients will be examined on first-generation dual-energy CT scanners (SOMATOM Definition Edge, Siemens Healthineers, Erlangen, Germany). The scanning ranges from the top of the diaphragm to the pubic symphysis. CT scanning parameters are as follows: tube voltage, 100 kVp (reference); effective tube current, 180 mAs (reference); rotation time, 0.5 s. Following non-contrast scanning, a non-ionic contrast agent will be administered intravenously. For patients > 60 kg of total body weight, 120 mL will be administered; for patients < 60 kg of total body weight, 100 mL will be administered (2.5–3.0 mL/s) (Table 1).

### 2.4. Data Collection

Intervention (T_int_):

- Antioxidant intervention: Drug (manufacturer, batch), dose, administration, supervising physician, time of completion, immediate adverse effects. Late adverse effects will be collected or actively investigated monthly by C.G.S.
- Placebo: Tablets having the same features as active ones. Drug (manufacturer, batch), dose, administration, supervising physician, time of completion, and immediate adverse effects. Late adverse effects will be collected or actively investigated monthly by C.G.S.

Clinical interview (T_ci_):

- Clinical history: Age, sex, occupation, allergies, immunizations, family history of diseases, comorbidities, surgical history, medication, supplementation, and hospital admissions. Pregnancies, births, and abortions.
- Habits: Physical activity (GPAQ), alcohol use (AUDIT), smoking status, history of recreational drugs. Sleep hygiene (Sleep Hygiene Index). Dietary questionnaire. History of exposure to toxic substances. Perceived Stress Scale 4 (PSS-4).
- Physical examination: Weight, height, abdominal circumference, body fat percentage, temperature, heart rate, systolic and diastolic arterial pressure.
- CE abdomen and pelvic CT indication: Acute disease history, referral physician, working diagnosis or CT indication, CT report.

Exposure (T_exp_):

- CT acquisition parameters: Time of non-contrast and CE phase CT acquisition; CT scanner manufacturer and model; scanning range; tube voltage and current; rotation time; contrast agent type, manufacturer, total volume and flow rate; saline type, manufacturer, total volume and flow-rate; portal phase delay time.
- Radiation dose estimation: CTDIvol; DLP; ED.

Post-intervention-like schedule: Imaging and laboratory tests are available in the electronic clinical records within 30 days before and after the exposure.

Post-study subjects’ protocol: Blood sample determinations: Blood samples #1 (T_base_) and #2 (T_post_), 10 mL each, will be collected for laboratory determination of the study outcomes.

- Oxidative stress laboratory determinations (secondary outcomes): Vitamin C (blood samples #1 and #2 for groups A_NONE-NONE_ and B_NONE-EXP_, and blood samples #1, #2, and # for groups C_PLAC-EXP_ and D_VITC-EXP_). Plasma FRAP, malondialdehyde (MDA), and F_2_-isoprostane levels. Laboratory determinations will be performed by trained personnel under supervision (A.G. and R.R.).
- DNA damage laboratory determination (primary outcome): Immunofluorescence analyses of isolated peripheral lymphocytes incubated with a specific γ-H2AX antibody. Laboratory determinations will be performed by trained personnel under supervision (M.M.).

### 2.5. Data Storage

The Data Monitoring Committee will be composed of C.G.S., C.G., B.R. and D.C (head). Data will be anonymized and stored in the cloud-based data center SASIBA, with limited access to the researchers (in particular, access to the randomization spreadsheet will be granted only to D.C.). SASIBA is a service generated by the Data Unit of the Center for Medical Informatics and Telemedicine of the Faculty of Medicine, which is available to the local scientific community. This Server (Hosting, VPS, Housing) will be used as a local Dropbox (OwnCloud) with automatic data backup (Oops!Backup Windows, TimeMachine Mac). This data center is housed in the central data center of the University of Chile, under the same standard of care as the sensitive data of the University, following physical security policies equivalent to TIER-2/3. The Dell Unity equipment follows multiple standards, including the Health Insurance Portability and Accountability Act of 1996 (HIPAA), and the core Vmware software also supports HIPAA.

### 2.6. Statistical Analysis

Continuous variables will be summarized as mean values ± standard deviations if normally distributed and compared with Student’s *t*-tests. Continuous variables will be summarized as median (interquartile ranges) if they are non-normally distributed and compared with Mann–Whitney U-tests. Dichotomous variables will be summarized as percentages and compared using Chi-squared tests. ANOVA tests will be performed to compare changes among the three subgroups. Box and whisker plots will be built to illustrate differences in OS and DNA damage biomarkers. Spearman and linear regression analyses will be performed to evaluate correlations and multivariate associations. Multivariate logistic regression analyses will be performed to evaluate determinants of γ-H2AX foci change. To examine whether the potential association of IR doses with γ-H2AX foci change is mediated by OS biomarkers, mediation analyses will be performed with the method described by Preacher and Hayes [61,62], which allows for testing the significance and magnitude of mediation. In all analyses, a two-sided significance level of *p* < 0.05 will be considered statistically significant. Data will be analyzed using IBM SPSS software version 29 (SPSS Inc., Chicago, IL, USA), STATA 18 (STATA Corp., College Station, TX, USA), and R version 4.3.1 (R Foundation for Statistical Computing, Vienna, Austria).

## 3. Discussion

This study is a limited, academic, randomized, placebo-controlled, four-arm clinical trial designed to evaluate the intervention consisting of an oral dose of vitamin C to counteract OS-mediated DNA damage in the scenario of exposure to IR-associated two-phase CE abdomen and pelvis CT.

The endpoints of the study are: (1) To compare the pre- and post-exposure change on OS and DNA-damage biomarkers in peripheral blood between exposed (not recipients of the oral vitamin C antioxidant intervention) and not exposed subjects (at an equivalent interval time); (2) To compare the pre- and post-exposure change on OS and DNA-damage biomarkers in peripheral blood among exposed patients, between recipients of a placebo, recipients of the oral vitamin C antioxidant intervention and not recipients of an intervention; (3) To estimate the mediating effect of IR-induced OS on DNA-damage biomarkers in peripheral blood of exposed subjects; and (4) To compare the total mediating effect of OS on IR-induced DNA damage among exposed subject, between recipients and not recipients of the oral vitamin C antioxidant intervention.

Beyond previous in vitro or mixed in vivo/in vitro studies [53], to the best of our knowledge, this is the first clinical outpatient study to be expressly designed to evaluate both (1) the preliminary assumption that OS plays a significant mediator role in IR-induced DNA damage; and (2) the hypothesis that enhancing the antioxidant capacity previous to exposure through an easy-to-use oral antioxidant intervention may counteract the associated IR-induced DNA damage through counterbalancing the OS-mediating effect on total DNA damage increase. 

Previous clinical studies [51,54] have investigated the hypothesis that an antioxidant intervention may counteract the IR-induced DNA damage without studying OS biomarkers to quantitatively size the mediating effect through this pathway, limiting further understanding of the underlying mechanisms and the overall potential of antioxidant intervention strategies. This is an important distinct methodological feature to aid in proposing further novel approaches. Compared to the study by Stehli et al. [51], which randomized patients to receive an intravenous antioxidant intervention, the current study aims to evaluate a simple oral intervention that may be used routinely in the outpatient setting, further making it unique in terms of its potential clinical impact. Also, the current study is centered on a clinical imaging examination with the most IR patient dose burden according to current international radiation dose estimation data, which is a strength compared to most previous studies in terms of the potential impact of its findings. Departing from a previous clinical outpatient study by Tao et al. [54], the current study proposes the use of patient dose estimates to allow for a comparison of its results with that of further studies on different IR-based clinical imaging examinations associated with stochastic effects in risk estimation fashion according to international recommendations. 

Furthermore, this is the first study to be performed in a multiple-group setting, with either exposure or no exposure, receiving either placebo, the antioxidant intervention or no intervention, each with appropriate statistical power, which is another strength of this study protocol to advance currently available literature. It should also be underscored that our measurements of baseline vitamin C will provide data for the first time to better characterize the clinical need for vitamin C supplementation to counterbalance IR-induced DNA damage. We also expect that the design and execution by a multidisciplinary team will allow a better understanding and more precise quantification of the role of OS mechanisms on the pathways that lead to IR-associated DNA damage, opening new avenues for potential counteracting effects by antioxidant interventions. 

The main limitations of our study are the use of a single CT protocol, a single antioxidant strategy, and a single-center design. Although current literature could not consistently support the hypothesis that a combination of antioxidants may provide an additive effect to vitamin C [52], whether a combination of antioxidants could offer better protection in particular clinical scenarios remains to be explored in further studies. We also acknowledge the use of a single DNA damage measurement method, whereas analyzing phosphorylation of ATM/ATR, p53, or cell death could provide a more detailed understanding and may be warranted in further studies. Lastly, while the current study is focused on the immediate effects of IR-induced OS mechanisms mediating DNA damage, the ultimate goal of advancing knowledge in this field is to provide the theoretical and evidence-based background that allows the study of potential long-term effects, e.g., whether the expected immediate effects may lower the risk of cancer among other, which remains as the follow-up research task to appropriately design with the results of the current study.

Computed tomography continues to be a matter of wide attention because of reports suggesting associated cancer risk, unexpected overexposures, over-utilization or inappropriate use, which heighten interest in making CT safer [63,64]. Reaction to these concerns has come from stakeholders, manufacturers, medical physicists, researchers and users, yet recent studies are again raising concerns related to these risks for a large number of patients [64,65]. Traditionally, the overuse of CT has often been attributed as the primary cause. However, recent data is derived from studies conducted in institutions with effective control mechanisms. In these settings, CT requests are filtered through a clinical decision support system, the typical CT doses are well below the national benchmark, and clinicians assess the CT indication appropriateness at outstanding high levels [64,65]. This study deals with the need expressed by the growing body of literature that leads to the conclusion that there is a need to increase awareness about potential opportunities focused on the opposing/receiving end of medical IR, particularly the defense system of patients against IR exposure. Further research is needed on novel intervention strategies to enhance the antioxidant capacity against IR-induced DNA damage. 

In conclusion, this is the first study designed to determine the mediating effects of IR-induced OS on DNA damage and the counteracting effect in recipients of an oral vitamin C intervention prior to IR exposure in the clinical scenario of outpatients undergoing two-phase CE abdomen and pelvis CT. Potentially favorable results of this study will generate the basis for larger clinical trials to further explore novel antioxidant strategies to counteract OS-mediated DNA damage in the scenario of exposure to IR-associated medical imaging examinations.

## Figures and Tables

**Table 1:** CT scanning parameters.

Parameters	Non-Contrast Phase	Contrast-Enhanced Phase
kV (reference)	100	100
mAs (reference)	180	180
Dose optimization level	3	7
Rotation time (s)	0.5	0.5
Delay (s)	2	80
Pitch	1	1
Collimator (mm)	128 × 0.6	128 × 0.6

## Data Availability

The datasets generated and/or analyzed during the current study are available through the corresponding author upon reasonable request. Study results will be disseminated at international conferences and published in peer-reviewed scientific journals. Camilo G. Sotomayor (MD, PhD) is responsible for replying to public and scientific queries (contact telephone number +562-2978-8412, e-mail camilosotomayor@uchile.cl, and address Dr. Carlos Lorca Tobar 999, University of Chile Clinical Hospital, Radiology Department, Santiago 8380453, Chile).

## Supplementary Materials

The following supporting information can be downloaded at: https://www.mdpi.com/article/10.3390/jcm13133866/s1, Table S1: Experimental studies of vitamin C-based antioxidant interventions to reduce ionizing radiation-induced DNA damage.
