# Supplementary material for: Ionizing Radiation-Induced Oxidative Stress in Computed Tomography—Effect of Vitamin C on Prevention of DNA Damage: PREVIR-C Randomized Controlled Trial Study Protocol
Source: J Clin Med. 2024 Jun 30;13(13):3866. doi: 10.3390/jcm13133866 (PMC11242585; doi:10.3390/jcm13133866)
Supplement: Supplementary file 1 [file jcm-13-03866-s001.zip › jcm-3001578-supplementary.pdf]

Study Protocol

Ionizing Radiation-Induced Oxidative Stress in Computed Tomography—Effect of Vitamin C on Prevention of DNA Damage: PREVIR-C Randomized Controlled Trial Study Protocol

Camilo G. Sotomayor [1,2,*], Camila González [3], Miki Soto [4], Nicolás Moreno-Bertero [4], Claudina Opazo [4], Baltasar Ramos [4], Gonzalo Espinoza [1], Álvaro Sanhueza [1], Gonzalo Cárdenas [1], Sebastián Yévenes [1], Jorge Díaz-Jara [1], José de Grazia [1], Marcia Manterola [5], Daniel Castro [1], Abraham A. I. J. Gajardo [6,7] and Ramón Rodrigo [8]

[1] Radiology Department, University of Chile Clinical Hospital, University of Chile, Santiago 8380420, Chile
[2] Anatomy and Developmental Biology Program, Institute of Biomedical Sciences, Faculty of Medicine, University of Chile, Santiago 8380453, Chile
[3] Faculty of Medicine, University of Santiago Chile, Santiago 9170022, Chile
[4] School of Medicine, Faculty of Medicine, University of Chile, Santiago 8380453, Chile
[5] Human Genetics Program, Institute of Biomedical Sciences, Faculty of Medicine, University of Chile, Santiago 8380453, Chile
[6] Intensive Care Unit, Medicine Department, University of Chile Clinical Hospital, University of Chile, Santiago 8380420, Chile
[7] Program of Pathophysiology, Institute of Biomedical Sciences, Faculty of Medicine, University of Chile, 8380453 Santiago, Chile
[8] Molecular and Clinical Pharmacology Program, Institute of Biomedical Sciences, Faculty of Medicine, University of Chile, Santiago 8380000, Chile
* Correspondence: camilosotomayor@uchile.cl; Tel.: +56-229-788-412

Table S1. Experimental studies of vitamin C-based antioxidant interventions to reduce ionizing radiation-induced DNA damage

Study model	Experimental method		Radiation			DNA-damage				Reference
	**Antioxidant con-centration/dose**	**Administration route**	**Sample**	**Antioxidant to exposure time**	**Dose**	**Study method**	**Significant reduction?**	**Dose dependent?**	**Time dependent?**	
***In-vitro* studies in human lymphocytes**	60 - 120 µM	Cell culture	*In vitro*	3 hr	350 mGy	γ-H2AX	Yes	Yes	NE	Xiao *et al*. 2014
	16 - 16000 µg/mL	Cell culture	*In vitro*	15 min – 4 hr	10 mGy	γ-H2AX	Yes	Yes	Yes	Brand *et al*. 2015
	0.88 - 88 µg/mL	Cell culture	*In vitro*	37 sec	50 mGy	γ-H2AX	Yes	Yes (Between 1/10 SC and 1 SC)	Yes	Bicheru *et al*. 2020
**Mixed *in-vitro/in-vivo***	600 mg	Oral	*In vitro/in-vivo*	1-2-3 hr	2000 mGy	Micronuclei	Yes	NE	Yes, best at 1 hr	Rostami *et al*. 2016
**Clinical studies**	1000 mg	Oral	*In vivo*	0.5-2 hr	~1400 mGy*cm	γ-H2AX	Yes	NE	No	Tao *et al*. 2019
**Randomized, double-blind placebo-controlled trials**	3000 mg	IV	*In vivo*	Minutes (?)	0 – 2 – 29 mSv	γ-H2AX	Yes (in the higher radiation-dose group)	Radiation-dose dependent effect	NE	Stehli *et al*. 2014

IV, intravenous; NE, not evaluated.

References

1. Bicheru, N. S., Haidoiu, C., Calborean, O., Popa, A., Porosnicu, I., & Hertzog, R. (2020). Effect of different antioxidants on X-ray induced DNA double-strand breaks using γ-H2AX in human blood lymphocytes. Health Physics, 119(1), 101-108.
2. Brand, M., Sommer, M., Ellmann, S., Wuest, W., May, M. S., Eller, A., ... & Uder, M. (2015). Influence of different antioxidants on X-ray induced DNA double-strand breaks (DSBs) using γ-H2AX immunofluorescence microscopy in a preliminary study. PLoS One, 10(5), e0127142.
3. Rostami, A., Moosavi, S. A., Moghadam, H. D., & Bolookat, E. R. (2016). Micronuclei assessment of the radioprotective effects of melatonin and vitamin C in human lymphocytes. Cell Journal (Yakhteh), 18(1), 46.
4. Stehli, J., Fuchs, T. A., Ghadri, J. R., Gaemperli, O., Fiechter, M., & Kaufmann, P. A. (2014). Antioxidants Prevent DNA Double-Strand Breaks From X-Ray–Based Cardiac Examinations: A Randomized, Double-Blinded, Placebo-Controlled Trial. Journal of the American College of Cardiology, 64(1), 117-118.
5. Tao, S. M., Zhou, F., Schoepf, U. J., Fischer, A. M., Giovagnoli, D., Lin, Z. X., ... & Zhang, L. J. (2019). The effect of prophylactic oral vitamin C use on DNA double-strand breaks after abdominal contrast-enhanced CT: A preliminary study. European Journal of Radiology, 117, 69-74.
6. Xiao, L., Tsutsui, T., & Miwa, N. (2014). The lipophilic vitamin C derivative, 6-o-palmitoylascorbate, protects human lymphocytes, preferentially over ascorbate, against X-ray-induced DNA damage, lipid peroxidation, and protein carbonylation. Molecular and cellular biochemistry, 394, 247-259.
